# Plant Translation Elongation Factor 1Bβ Facilitates *Potato Virus X* (PVX) Infection and Interacts with PVX Triple Gene Block Protein 1

**DOI:** 10.1371/journal.pone.0128014

**Published:** 2015-05-28

**Authors:** JeeNa Hwang, Seonhee Lee, Joung-Ho Lee, Won-Hee Kang, Jin-Ho Kang, Min-Young Kang, Chang-Sik Oh, Byoung-Cheorl Kang

**Affiliations:** 1 Department of Plant Science, Plant Genomics & Breeding Institute, and Research Institute of Agriculture and Life Sciences, Seoul National University, Seoul, 151–921, Korea; 2 Korea Institute of Science and Technology Information, Seoul, 130–741, Korea; 3 Crop Biotechnology Institute/GreenBio Science and Technology, Seoul National University, Pyeongchang, 232–916, Korea; 4 Department of Horticultural Biotechnology, Kyung Hee University, Yongin, 446–701, Korea; University of California, Riverside, UNITED STATES

## Abstract

The eukaryotic translation elongation factor 1 (eEF1) has two components: the G-protein eEF1A and the nucleotide exchange factor eEF1B. In plants, eEF1B is itself composed of a structural protein (eEF1Bγ) and two nucleotide exchange subunits (eEF1Bα and eEF1Bβ). To test the effects of elongation factors on virus infection, we isolated *eEF1A* and *eEF1B* genes from pepper (*Capsicum annuum*) and suppressed their homologs in *Nicotiana benthamiana* using virus-induced gene silencing (VIGS). The accumulation of a green fluorescent protein (GFP)-tagged *Potato virus X* (PVX) was significantly reduced in the *eEF1Bβ*- or *eEF1Bɣ*-silenced plants as well as in *eEF1A*-silenced plants. Yeast two-hybrid and co-immunoprecipitation analyses revealed that eEF1Bα and eEF1Bβ interacted with eEF1A and that eEF1A and eEF1Bβ interacted with triple gene block protein 1 (TGBp1) of PVX. These results suggest that both eEF1A and eEF1Bβ play essential roles in the multiplication of PVX by physically interacting with TGBp1. Furthermore, using *eEF1Bβ* deletion constructs, we found that both N- (1-64 amino acids) and C-terminal (150-195 amino acids) domains of eEF1Bβ are important for the interaction with PVX TGBp1 and that the C-terminal domain of eEF1Bβ is involved in the interaction with eEF1A. These results suggest that *eEF1Bβ* could be a potential target for engineering virus-resistant plants.

## Introduction

Plant viruses are excellent subjects for the elucidation of host—microbe interactions because they are simple and obligate intracellular parasites and are entirely dependent on host cellular metabolism for their life cycle. Virus infection therefore involves interactions between viral components and host factors [1, 2], and mutation or absence of the appropriate host factors may result in resistance of plants against viruses [3].

Translation elongation in eukaryotes requires a set of non-ribosomal proteins called eukaryotic elongation factors (eEFs). eEF1 comprises eEF1A and eEF1B [4]. eEF1A is one of the most abundant proteins in eukaryotic cells and delivers aminoacyl-tRNA (aa-tRNA) to the elongating ribosome in a GTP-dependent manner. In addition to its role in peptide chain elongation, eEF1A has various other functions including in quality control of newly produced proteins, ubiquitin-dependent protein degradation, and organization of the actin cytoskeleton [5–7]. Moreover, there have been numerous reports that eEF1A plays a pivotal role in the replication of RNA viruses by interacting with viral RNA and/or viral RNA-dependent RNA polymerase (RdRp) [8–14]. The interaction between eEF1A and the 3’-untranslated region (3’-UTR) of *Turnip yellow mosaic virus* (TYMV) RNA enhances the translation of TYMV RNA [13]. eEF1A from wheat (*Triticum* spp.) germ and *N*. *benthamiana* interacts with a pseudoknot (PK) structure upstream of a tRNA-like structure (TLS) in the *Tobacco mosaic virus* (TMV) genome [14]. eEF1A has been found to bind directly to a viral RdRp, VPg-protease (VPg-Pro) of *Turnip mosaic virus* (TuMV), and the methyltransferase (MT) domain of TMV RdRp [15–17]. Furthermore, in the cases of *Tomato bushy stunt virus* (TBSV), TMV, and *West Nile virus*, eEF1A binds to both the viral RNA and the viral RdRp [4, 11, 18, 19]. Mutations in eEF1A lead to a decrease in both minus strand synthesis of the *West Nile virus* genome [11] and accumulation of TBSV RNA [19].

The eEF1B protein is a guanine nucleotide exchange factor that binds to GDP-bound eEF1A. Different subunits constitute eEF1B depending on the species. In yeast, eEF1B is made of two subunits: a guanine nucleotide exchange protein (eEF1Bα) and a structural protein (eEF1B*γ*). In higher eukaryotes, the eEF1B complex has a second nucleotide exchange factor (eEF1Bβ or eEF1Bδ). Plant eEF1B is composed of a structural protein (eEF1B*γ*) and two nucleotide exchange subunits (eEF1Bα and eEF1Bβ), whereas the metazoan complex is a heteromer of at least four subunits: a structural protein (eEF1B*γ*), two exchange factors (eEF1Bα and eEF1Bδ), and a unique tRNA synthetase (valine-tRNA synthetase) [4, 18]. Previously, we showed that *Capsicum* eEF1Bβ is required for infection of TMV and interacts with the MT domain of TMV RdRp as well as eEF1A [15]. In addition, the eEF1B*γ* subunit of yeast was shown to be involved in TBSV minus-strand synthesis with eEF1A [19]. Together, these reports suggest that eEF1B could play an important role in viral multiplication. The involvement of eEF1A in infection has been well studied for several viruses [8–14], whereas the precise roles of each eEF1B subunit in viral multiplication still remain unknown.

*Potato virus X* (PVX) belongs to the *Potexvirus* genus and consists of a positive-sense single-stranded RNA of about 6500 nucleotides encoding five proteins [20]. Among its five open reading frames (ORFs) [21], the product of ORF1, the viral replicase, is responsible for RNA synthesis. The triple gene block (TGB) proteins [TGBp1 (TGB25K), TGBp2 (TGB12K), and TGBp3 (TGB8K)] encoded by the internal overlapping reading frames are necessary for virus cell-to-cell and long-distance movement [22, 23]. ORF5 encodes the coat protein (CP), which is necessary for encapsidation and virus movement [24]. PVX has been used as a model system for studying RNA silencing and plant immune responses as well as virus movement [25].

In this study, we identified each subunit of eEF1B from pepper (*C*. *annuum*) and studied their effects on PVX infection in *N*. *benthamiana*. We found that the accumulation of PVX was specifically reduced in *eEF1Bβ*- or *eEF1Bγ*-silenced plants as well as *eEF1A*-silenced plants. Using yeast two-hybrid and co-immunoprecipitation analyses, we demonstrated that eEF1A interacts with eEF1Bα and eEF1Bβ, and that each eEF1A and eEF1Bβ interacts with the TGBp1 protein of PVX. Together with previous results, our findings suggest that eEF1Bβ could be a host factor for PVX infection and that interaction between eEFs and viral components is commonly required for plant RNA virus multiplication.

## Materials and Methods

### Identification of Sequences for Subunits of *eEF1B* and Phylogenetic Analysis

The sequences of *eEF1B* subunits from five species [Arabidopsis (*A*. *thaliana*), rice (*O*. *sativa*), tomato (*S*. *lycopersicum*), pepper (*C*. *annuum*), and *N*. *benthamiana*] were identified from the National Center for Biotechnology Information (NCBI, http://www.ncbi.nlm.nih.gov/), Sol Genomics Network database (SGN, http://solgenomics.net/) and *Capsicum annuum* database (http://peppergenome.snu.ac.kr). Using the unique features of each subunit in rice [4], we searched for interpro IDs of each eEF1B subunit in Arabidopsis and used them to search for each subunit of eEF1B in tomato and pepper. In the case of *eEF1Bα* in pepper, only partial sequences were retrieved from the NCBI. To obtain full-length sequences of pepper *eEF1Bα*, 3’ rapid amplification of cDNA ends (3’ RACE)-PCR was performed using cDNA of pepper ‘Early Carl Wonder (ECW)’ and oligonucleotide primers (1Bα-3UTR and 3'-RACE(AP) primers, S1 Table) as described previously [26]. Sequences of *eEF1Bβ* and *eEF1Bγ* in pepper ‘CM334’ were obtained from the *Capsicum annuum* database (http://peppergenome.snu.ac.kr). The cDNA sequences of *eEF1Bβ* and *eEF1Bγ* in pepper ‘ECW’ were amplified using specific primers (eEF1B beta and gamma primers, S1 Table). Based on homology searches, *eEF1Bα* and *eEF1Bβ* in *N*. *benthamiana* were obtained from SGN and *eEF1Bγ* in *N*. *benthamiana* was obtained from NCBI.

The amino acid sequences of *eEF1B* subunits were aligned by Clustal W. Phylogenetic analysis was performed using the neighbor-joining method to construct phylogenetic tree (MEGA version 5.1 software; [27]).

### Plasmid Construct for Virus-Induced Gene Silencing (VIGS)

The cDNA sequences of *N*. *benthamiana* genes homologous to pepper were amplified using Ex Taq DNA polymerase (TaKaRa, Shiga, Japan) and oligonucleotide primers (eEF1A-LIC and eEF1B alpha-, beta-, gamma-LIC primers, S1 Table). Primers were designed using the gene coding region to specifically amplify each gene. In the case of *eEF1Bα* and *eEF1Bβ*, silencing primers were designed to amplify the region encoding the N-terminus, which is more divergent than the region encoding the C-terminus. Modified ligation-independent cloning was used for high-throughput cloning into the TRV VIGS vector [28]. All PCR products were purified using DNA clean & Concentrator-100 (Zymo Research, Orange, USA). The purified PCR products (15 fmol) were treated with T4 DNA polymerase (LIC qualified, Novagen, San Diego, CA, USA) in 10x reaction buffer containing 5 mM dATP at 22°C for 30 min followed by inactivation of the T4 DNA polymerase for 20 min at 70°C. The TRV2-LIC vector was digested with *PstI* and treated with T4 DNA polymerase using dTTP instead of dATP. A total of 22.5 fmol treated PCR product and TRV2-LIC vector was mixed and incubated at 65°C for 2 min, and further incubated at 22°C for 10 min. Then, 3 μL of the mixture was transformed into *E*. *coli* DH10B competent cells. Transformants were tested by PCR amplification using TRV-LIC insert primers. Plasmids from positive clones were purified (Zymo Research, USA). Sequencing analysis was performed at the National Instrumentation Center for Environmental Management (Seoul National University, Seoul, Korea).

### Plant Materials and *Agrobacterium* Infiltration

*N*. *benthamiana* plants were grown at 25°C in a growth chamber with a 16-h light/8-h dark cycle. For the VIGS experiment, the TRV VIGS system was used [29][30]. Briefly, pTRV1 or pTRV2 and its derivatives were introduced into cells of *Agrobacterium tumefaciens* strain GV2260. *Agrobacterium* cultures were grown overnight at 28°C in Luria-Bertani (LB) medium containing antibiotics (50 mg/L kanamycin and 50 mg/L rifampicin). *Agrobacterium* cells were harvested and resuspended in infiltration medium (10 mM MgCl_2_, 10 mM MES, 200 μM acetosyringone), adjusted to 0.4 OD_600_, and incubated at room temperature for at least 3 h. *Agrobacterium* carrying pTRV1 was mixed in a 1:1 ratio with pTRV2 or its derivatives and infiltrated into leaves of *N*. *benthamiana*.

### RNA Extraction and Real-Time PCR Analysis

Total RNA was extracted from leaves of *N*. *benthamiana* using an RNeasy plant minikit (Qiagen, Hilden, Germany). First-strand cDNA was synthesized using 2 μg total RNA, oligo dT primers and M-MLV reverse transcriptase (Promega, Madison, USA) according to the manufacturer’s protocol. The expression levels of each gene in VIGS plants were monitored at 13 dpi by real-time PCR using gene-specific primers (eEF1A-RT and eEF1B alpha-, beta-, gamma-RT primers) that anneal outside the targeted silencing region (S1 Table). The real-time PCR was performed using a Rotor-gene Q real-time PCR cycler (Qiagen, USA). Thermal cycling was as follows: denaturing at 95°C for 5 min, followed by 50 cycles of denaturing at 95°C for 1 min, annealing at 53°C (*eEF1Bα* and-*eEF1Bγ*), 56°C (*eEF1Bβ*), or 58°C (*actin*) for 1 min and extension at 72°C for 1 min [31].

### Virus Infection and Evaluation of Resistance

PVX-GFP inocula were prepared from leaves of *N*. *benthamiana* plants that had been inoculated with *Agrobacterium* containing pSPVX-sGFP [20, 32]. Plants were inoculated at the four- to six-leaf stage. Carborundum was lightly applied to the two oldest leaves, followed by rub-inoculation with virus produced by grinding systemically infected *N*. *benthamiana* tissue in 100 mM potassium phosphate buffer, pH 7.0 (1 g tissue: 10 mL buffer). Mock- and non-inoculated controls were included. After inoculation, the plants were monitored daily for symptom development.

Leaf tissue was tested for the presence of virus using DAS-ELISA according to the manufacturer’s instructions (Agdia, Inc., Elkhart, USA). Virus accumulation was tested at 5 days post inoculation (dpi) for inoculated and upper non-inoculated leaves. GFP was also visualized using a confocal scanning microscopy (LSM 510; Carl Zeiss, Jena, Germany) at 7 dpi.

### Yeast Two-Hybrid Analysis

Yeast transformation and analyses were performed using the ProQuest Two-Hybrid System with Gateway Technology (Invitrogen, http://www.invitrogen.com). Using PCR, full-length *eEF1A* and *eEF1Bβ* were amplified from *C*. *annuum* ‘ECW’ cDNA. The PVX-encoded proteins were amplified from PVX (UK)-infected *N*. *benthamiana*. ProQuest yeast two-hybrid vectors pDEST22 containing the *Capsicum eEF1A* or *eEF1Bβ*, and pDEST32 containing PVX genes were transformed into the yeast strain MaV203 (ProQuest; Invitrogen, http://www.invitrogen.com). Yeast transformants were plated on synthetic complete medium (SC) lacking leucine (Leu) and tryptophan (Try). After 72 h, large colonies were picked and cultured in SC liquid medium lacking Leu and Try. One day later, 10 μL cultured cells was applied to selection plates [SC medium lacking Leu, Try, histidine (His) with 10 or 25 mM 3-amino-1, 2, 4-triazole (3AT)] to examine protein interactions.

### Bimolecular Fluorescent Complementation (BiFC) Assay

To construct binary plasmids, sequences encoding the full-length of eEF1A, eEF1Bβ, and PVX TGBp1 was fused to those for either the N-terminal fragment of YFP in pSPYNE-35S (YN) or the C-terminal fragment of YFP in pSPYCE-35S (YC) [33], generating eEF1A-YC, eEF1B-YN, PVX TGBp1-YC, PVX TGBp1-YN. These constructs were transformed into *A*. *tumefaciens* strain GV2260, which was then grown on LB media with 50 mg/L kanamycin and 50 mg/L rifampicin for 1 d. The transformed *A*. *tumefaciens* GV2260 were harvested, suspended in infiltration buffer (10 mM MES, 10 mM MgCl_2_, and 200 μM acetosyringone) to an optical density at 0.4 O.D_600_, then incubated at room temperature for 3 h and infiltrated into *N*. *benthamiana* leaves using a syringe without a needle. Yellow fluorescent protein (YFP) fluorescence was analyzed 36 h–48 h after agro-infiltration using a confocal scanning microscope (Carl Zeiss, LSM710).

### Co-Immunoprecipitation (Co-IP) Assay

Proteins were transiently co-expressed by *Agrobacterium* infiltration in leaves of *N*. *benthamiana*. *C*. *annuum* eEF1A and eEF1Bβ were expressed using HA-tagged pEarlyGate (pEG) 201 vector [34], and the CMV 2b and PVX TGBp1 proteins were expressed using FLAG-tagged pEG202 vector [34], respectively. Co-IP assays were performed as described previously [35]. Briefly, leaves were harvested 2 d after infiltration, and total protein was extracted with an extraction buffer [GTEN: 10% glycerol, 25 mM Tris (pH 7.5), 1 mM EDTA, 150 mM NaCl, 10 mM DTT, 0.1% Triton X-100, 1X plant protease inhibitor (Sigma-Aldrich), 1X phosphatase inhibitor (Sigma), and 2% w/v polyvinypolylpyrrolidone]. Protein extracts were incubated with HA tag antibody-agarose beads for IP from 6 h to overnight. Finally, beads were collected and washed six times with an IP buffer (GTEN containing 0.15% Nonidet P-40 and 1 mM DTT). The bead-bound proteins were eluted with 5 × SDS sample buffer [10% w/v SDS, 10 mM beta-mercaptoethanol, 20% v/v glycerol, 0.2M Tris-HCl (pH 6.8), 0.05% w/v bromophenol blue] and denatured by boiling at 95°C for 5 minutes. The denatured proteins were separated via 12% SDS-PAGE and immunoblotted with anti-HA antibody (Sigma) and anti-FLAG antibody (Sigma).

### Construction of Deletion Mutants of *eEF1Bβ*

*eEF1Bβ* of *C*. *annuum* ‘ECW’ consisted of 696 nucleotides. *eEF1Bβ* variants without the N-terminal region encoded by nucleotides 1–192 (eEF1BβΔN, lacking amino acids 1–64), middle region encoded by nucleotides 193–300 (eEF1BβΔM, lacking amino acids 65–100), or C-terminal region encoded by nucleotides 450–570 (eEF1BβΔC, lacking amino acids 150–190) were amplified by PCR from ECW and expressed using FLAG-tagged pEG202 vector.

## Results

### Classification of Plant *eEF1B* Proteins as α, β, and γ Subunits

**Fig 1:**
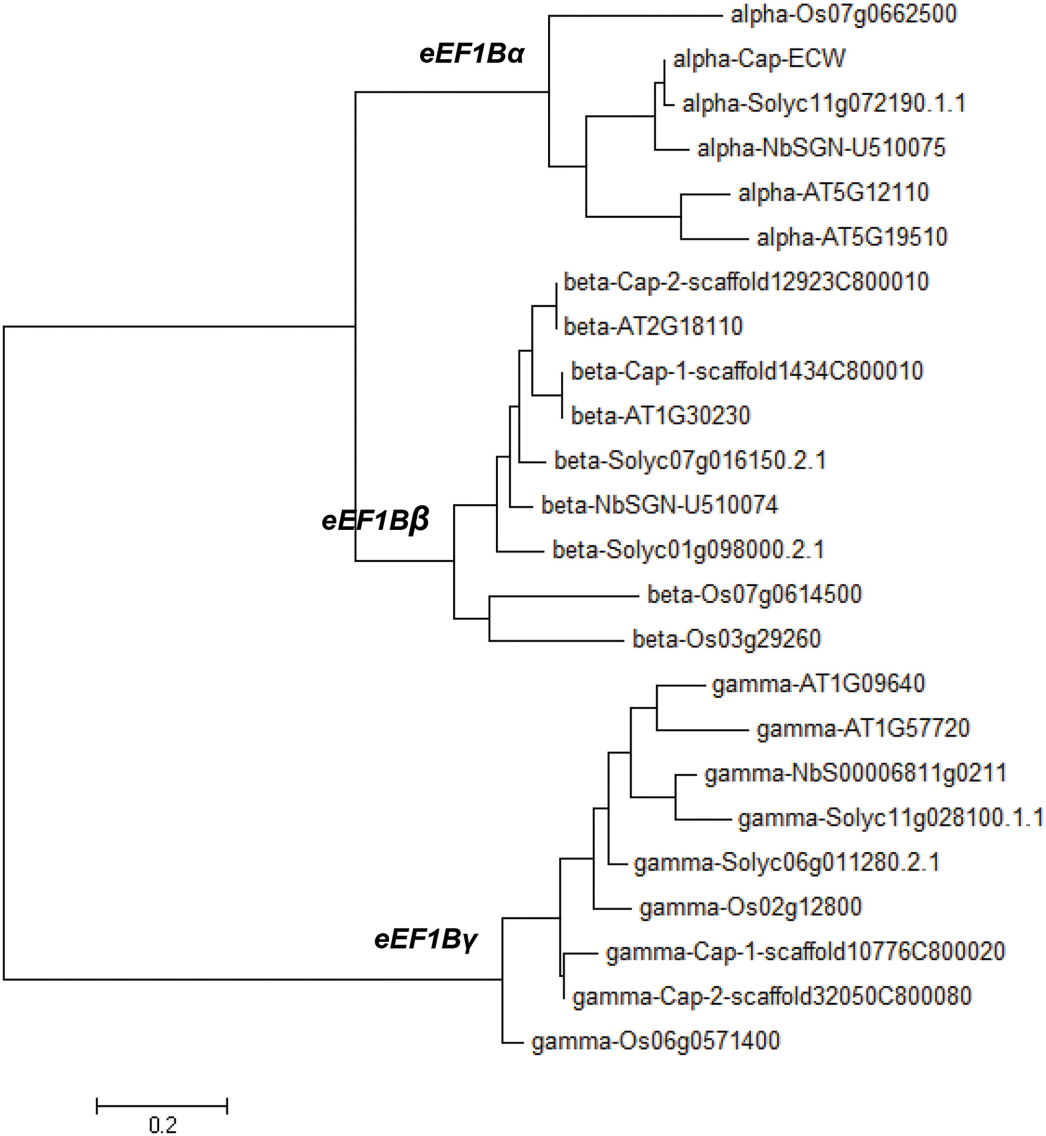
Phylogenetic tree of eEF1Bα, eEF1Bβ, eEF1Bγ proteins from five different plant species. The phylogenetic tree of deduced amino acid sequences was generated with the neighbor-joining method using MEGA5 software (http://www.megasoftware.net). Bootstrap values are from 1000 replicates, indicated above the nodes. Sequences of *A*. *thaliana*, *C*. *annuum*, *N*. *benthamiana*, *O*. *sativa* and *S*. *lycopersicum* proteins were obtained from NCBI (http://www.ncbi.nlm.nih.gov/), the SOL genome database (http://solgenomics.net) and the pepper genome database (http://peppergenome.snu.ac.kr).

In plants, eEF1B is composed of three subunits (α, β and γ) [4]. Although the involvement of eEF1Bβ and eEF1B*γ* in TMV and TBSV infection in *N*. *benthamiana* has been studied [15, 19], the exact roles of each eEF1B subunit in viral multiplication still remain unknown. To elucidate the roles of each subunit of eEF1B in virus infection, first we obtained gene sequences encoding eEF1Bα, eEF1Bβ, and eEF1Bγ subunits from Arabidopsis (*Arabidopsis thaliana*), rice (*Oryza sativa*), tomato (*Solanum lycopersicum*), pepper (*C*. *annuum*), and tobacco (*N*. *benthamiana*: Table 1). In rice, eEF1Bα and eEF1Bβ have a conserved phosphorylation site at casein kinase 2 (CK2, FG-(E/D)-E**T**EE: [4]), whereas only eEF1B*β* has a conserved putative cyclin-dependent kinase (CDK) phosphorylation site [^89^ **T**P–(P/S)–(V/A), as numbered in the rice sequence] just ahead of the CK2 site. eEF1B*γ* has two hydrophobic domains (domain I and II) and a CDK phosphorylation motif localized in the C-terminal region (domain II: [4]). Using the unique features of each subunit, we found that there are two copies each of *eEF1Bα*, *β* and *γ* in Arabidopsis, and one copy of *eEF1Bα* and two copies of *eEF1Bβ* and *eEF1Bγ* in rice and tomato (Table 1). Similarly, one copy of *eEF1Bα* and two copies of *eEF1Bβ* and *eEF1Bγ* were identified in pepper (Table 1). Although the exact copy number of each gene was not identified in *N*. *benthamiana* we have selected one contig from each gene that has the highest similarity to that of pepper (Table 1). Phylogenetic analysis of amino acid sequences of the eEF1B subunits from five different plant species supported the classification of the eEF1B subunits of pepper and *N*. *benthamiana* into three groups (*eEF1Bα*, *eEF1Bβ* and *eEF1Bγ*) as expected (Fig 1). In pepper, eEF1Bα and eEF1Bβ shared around 60% similarity at the amino acid sequence level (Table 2) and they had higher similarity in the C-terminal region than near the N-terminus (data not shown). There was no amino acid sequence similarity between *eEF1Bγ* and the other two subunits (*eEF1Bα* and *eEF1Bβ*) in pepper (Table 2). By contrast, the similarity between the two pepper copies of *eEF1Bβ* and *eEF1Bγ* was 91% and 78%, respectively (Table 2).

**Table 1:** Copy numbers of plant *eEF1B* subunits in five plant species.

Species	α-subunit		β-subunit		γ-subunit	
	copy number	sequence ID	copy number	sequence ID	copy number	sequence ID
*Arabidopsis thaliana*	2	AT5G12110 ^1^, AT5G19510 ^1^	2	AT1G30230 ^1^, AT2G18110 ^1^	2	AT1G09640 ^1^, AT1G57720 ^1^
*Oryza sativa*	1	Os07g0662500 ^1^	2	Os07g0614500 ^1^, Os03g29260 ^1^	2	Os02g12800 ^1^, Os06g0571400 ^1^
*Solanum lycorpersicum*	1	Solyc11g072190 ^1^	2	Solyc01g098000 ^1^, Solyc07g016150 ^1^	2	Solyc06g011280 ^1^, Solyc11g028100 ^1^
*Capsicum annuum*	1	AY480020 ^1^	2	PGAv.1.5.scaffold100 ^3^, PGAv.1.5.scaffold1125 ^3^	2	PGAv.1.5.scaffold117 ^3^, PGAv.1.5.scaffold546 ^3^
*Nicotiana benthamiana*	nd^4^	SGN-U510075 ^2^	nd^4^	SGN-U510074 ^2^	nd^4^	NbS00006811g0211 ^1^

^1^Sequence ID from NCBI database

^2^Sequence ID from Sol genome database.

^3^Sequence ID from pepper genome database (http://peppergenome.snu.ac.kr, v1.5 SCAFFOLD)

^4^ not determined

**Table 2:** Similarity among eEF1B subunits in *Capsicum annuum* at the amino acid level.

eEF1B subunits	α-subunit (AY480020)^1^		β-subunit (PGAv.1.5.scaffold100)^2^		β-subunit (PGAv.1.5.scaffold1125)^2^		γ-subunit (PGAv.1.5.scaffold117)^2^		γ-subunit (PGAv.1.5.scaffold546)^2^	
	Coverage (%)	Similarity (%)	Coverage (%)	Similarity (%)	Coverage (%)	Similarity (%)	Coverage (%)	Similarity (%)	Coverage (%)	Similarity (%)
α-subunit (AY480020)^1^	-	-	81	57	81	59	12	38	22	25
β-subunit (PGAv.1.5.scaffold100)^2^	-	-	-	-	100	91	none	none	none	none
β-subunit (PGAv.1.5.scaffold1125)^2^	-	-	-	-	-	-	none	none	none	none
γ-subunit (PGAv.1.5.scaffold117)^2^	-	-	-	-	-	-	-	-	100	78
γ-subunit (PGAv.1.5.scaffold546)^2^	-	-	-	-	-	-	-	-	-	-

^1^Full length sequence of *eEF1Bα* was obtained from *C*. *annuum* ‘ECW’ by 3’RACE-PCR based on the partial sequences of NCBI gene bank (AY480020).

^2^Sequences of *C*. *annuum eEF1Bβ* and *eEF1Bγ* were obtained from pepper genome database (http://peppergenome.snu.ac.kr, v1.5 SCAFFO

### *eEF1Bβ* and *eEF1Bγ* as well as *eEF1A* Are Important for PVX Infection

**Fig 2:**
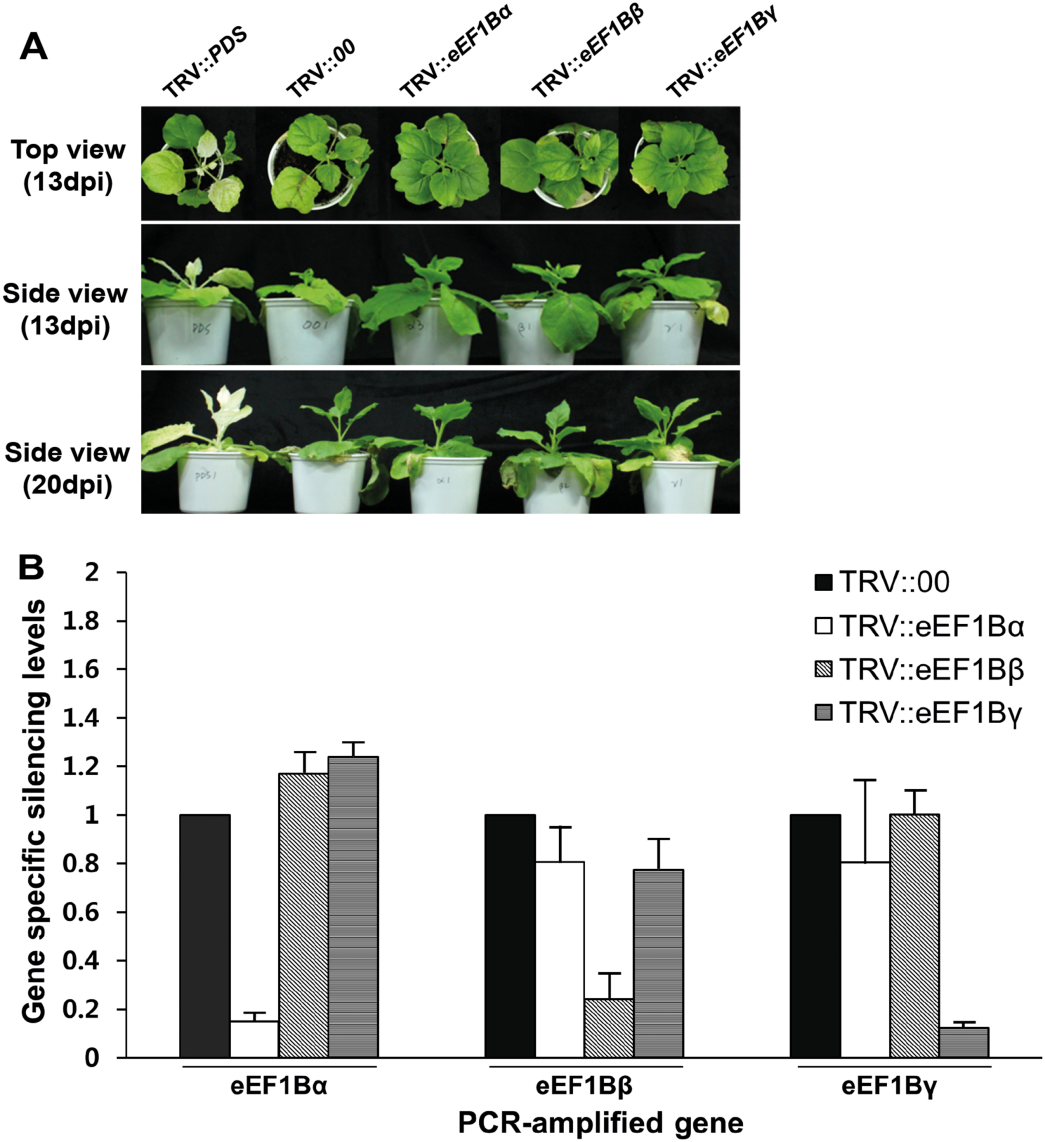
Silencing of *eEF1B* subunits in *N*. *benthamiana*. (A) Phenotype of *eEF1Bα*-, *β*- or *γ*-silenced *N*. *benthamiana*. TRV::*PDS* was used as a positive control for the VIGS experiment. The phenotype was compared with non-silenced plants (empty vector, TRV::00). Time points correspond to 13 and 20 days after TRV agro-infiltration. (B) Relative expression of each subunit in gene specific-silenced plants. The expression levels of each gene were determined 13 days after TRV agro-infiltration by quantitative RT-PCR and are given relative to the expression of TRV::00, which was set to 1. Relative levels were calculated using *actin* as a standard control. Bars represent standard errors for six biological replicates. Asterisks represent significant differences between TRV::00 and *eEF1B*-silenced plants (unpaired *t*-test: **P <0.01; ***P <0.001).

Previously, we found that pepper eEF1Bβ is involved in TMV infection and interacts with the MT domain of TMV RdRp [15], which was the first report regarding the roles of plant eEF1B in virus infection using plant eEF1B. To explore the roles of pepper *eEF1Bα*, *eEF1Bβ* and *eEF1Bγ* subunits in PVX infection, the genes encoding homologs of these subunits and *eEF1A* were silenced in *N*. *benthamiana* using a *Tobacco rattle virus* (TRV)-mediated VIGS method. We chose to use *N*. *benthamiana* for these experiments because it can be infected with large range plant viruses and also is highly amenable to VIGS [15]. In addition, *Capsicum* and *Nicotiana* are both members of the Solanaceae family and usually exhibit a high degree of similarity in gene coding sequences. We designed primers to specifically amplify each subunit but cover all copies within the same subunit from *N*. *benthamiana*. The resulting PCR fragments of *N*. *benthamiana eEF1Bα*, *eEF1Bβ*, and *eEF1Bγ* were 232, 241, and 166 bp, respectively. The amplified sequences from *N*. *benthamiana* showed over 90% similarity to those from pepper. These amplicons were used to prepare VIGS constructs to individually silence each subunit gene in *N*. *benthamiana*. The silencing phenotypes for each gene were observed at 13 and 20 days post infiltration (dpi; Fig 2A). TRV::*PDS* infiltrated plants were used as a positive control in the VIGS experiment. *N*. *benthamiana* plants silenced for *eEF1Bα*, *eEF1Bβ* or *eEF1Bγ* were developmentally similar to the plants infected with TRV empty vector (TRV::00) or TRV::*PDS* at 13 and 20 dpi. For example, all plants showed mild symptoms of virus infection and reduced growth (Fig 2A). However, *eEF1A*-silenced plants showed severe developmental defects and chlorotic leaves at 20 dpi (Fig A in S1 File). *eEF1Bα*-, *eEF1Bβ*- and *eEF1Bγ*-silenced plants showed reduced mRNA levels of the specific target genes without changes in the expression of the genes for the other subunits (Fig 2B), indicating the specific suppression of the target subunit gene and proper silencing of all copies of each subunit gene in the VIGS plants.

**Fig 3:**
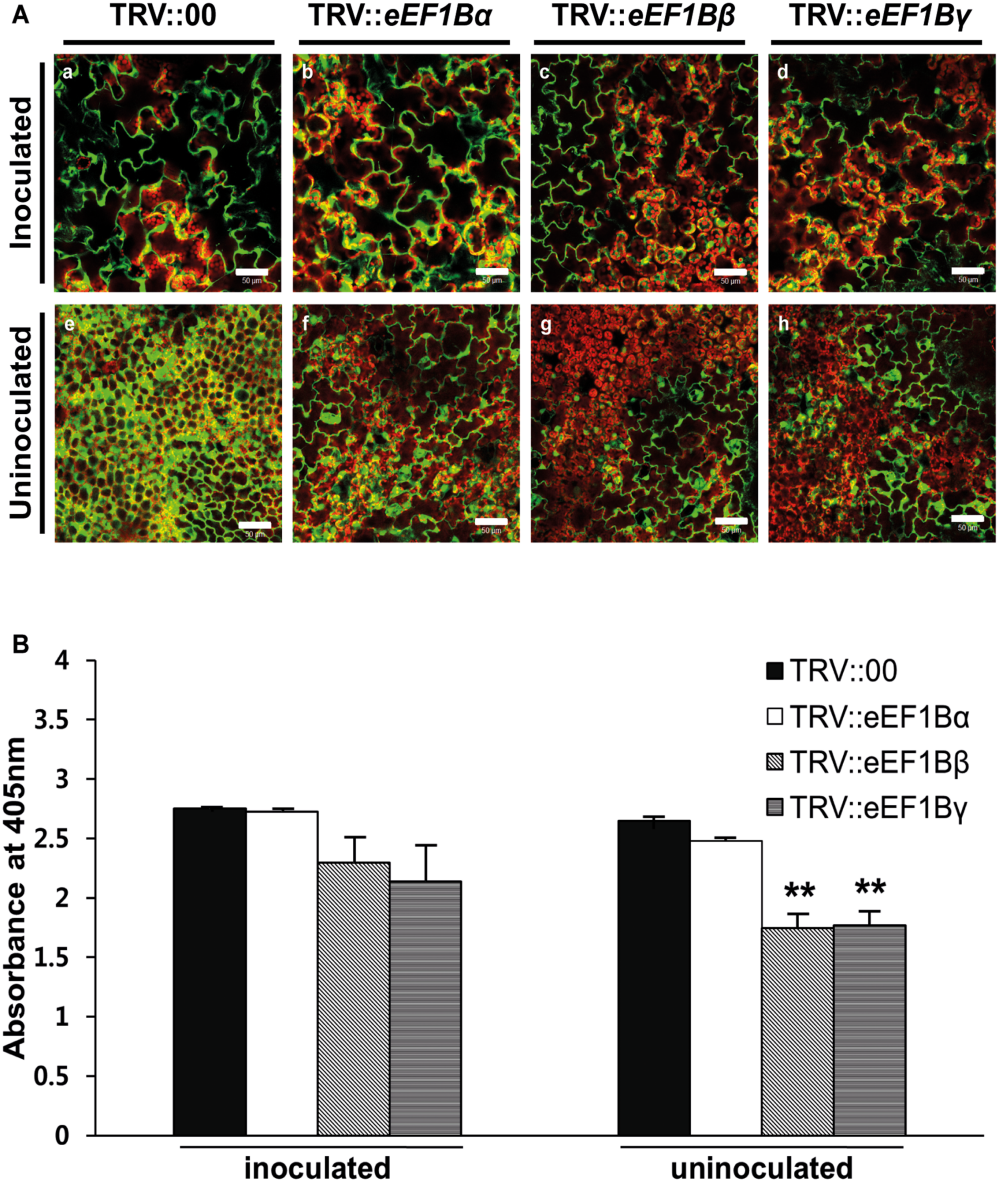
Effects of *eEF1Bα*-, *β*- or *γ*-silencing on PVX infection in *N*. *benthamiana*. (A) Spreading of PVX-GFP in *eEF1B* subunit-silenced plants and TRV::00 control plants. Fluorescence was visualized at 7 dpi in the inoculated (a-d) and uninoculated leaves (e-h). Green fluorescence signal indicates presence of GFP-fused PVX and chloroplasts are revealed by red autofluorescence. Scale bars = 50 μm. (B) Accumulation levels of PVX in gene-specific silenced plants. Accumulation of PVX in the inoculated and uninoculated leaves was tested by DAS-ELISA 5 days after PVX infection. This time point corresponds to 18 days after TRV agroinfiltration. Nine plants were tested in 3 independent repeats, with 3 biological plants for each treatment. The error bars indicate standard error. Asterisks denote significant differences between TRV::00 and *eEF1B*-silenced plants (unpaired *t*-test: **P <0.01).

To determine the effects of silencing each gene on PVX infection, PVX-GFP was used to inoculate the upper two leaves of VIGS plants, and virus multiplication was evaluated by observing the presence of GFP signal and the levels of PVX coat protein (CP) accumulation. In the inoculated leaves, all VIGS plants showed strong GFP signals at 7 dpi (Fig 3A, panels a to d). In the uninoculated upper (systemic) leaves, however, *eEF1Bβ*- and *eEF1Bγ*-silenced plants showed relatively weak GFP signals compared with TRV::00 and *eEF1Bα*-silenced plants (Fig 3A, panels e to h). To confirm these results, we measured the amount of PVX CP in inoculated leaves and uninoculated upper leaves at 5 dpi by ELISA. In the inoculated leaves, the *eEF1Bα*-silenced plants showed high levels of PVX CP equivalent to that in the TRV::00 plants (99% of the value in TRV::00 plants). PVX CP accumulation in the *eEF1Bβ*- and *eEF1Bγ*-silenced plants was slightly lower compared to that in the TRV::00 plants (84 and 78% of TRV::00 values; Fig 3B). In the uninoculated upper leaves, the *eEF1Bα*-silenced plants accumulated similar levels of PVX compared with TRV::00 plants (94% of TRV::00 values). However, the *eEF1Bβ*- and *eEF1Bγ*-silenced plants showed significantly reduced levels of PVX compared with TRV::00 plants (66 and 67% of TRV::00 values; Fig 3B). We also tested the effects of silencing *eEF1A* in *N*. *benthamiana*. Although silencing of *eEF1A* led to severe developmental defects at 20 days after agro-infiltration (Figs A and B in S1 File), strong inhibition of virus multiplication was observed in the inoculated leaves and uninoculated upper leaves 7 days after PVX infection (Fig C in S1 File). Taken together, these results indicate that *eEF1Bβ* and *eEF1Bγ* as well as *eEF1A* are required for PVX infection in plants.

### Interactions between eEF1A, eEF1B, and PVX-Encoded Proteins

**Fig 4:**
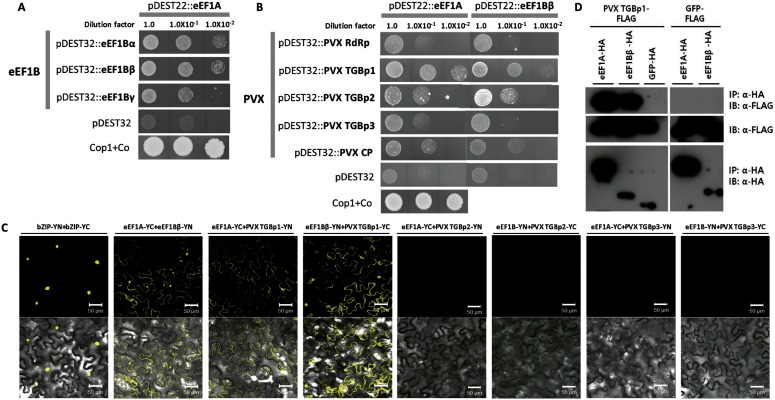
Protein-protein interaction between elongation factors and viral components. (A) Interaction between *C*. *annuum* eEF1A and eEF1B subunits in the yeast two-hybrid system. (B) Interaction between *C*. *annuum* eEF1s and PVX components in the yeast two-hybrid system. Individual transformants were grown on SC-Leu-Try-His+3AT (10 mM) plates for 9 (A) or 6 days (B). pDEST22::Cop1 and pDEST32::Co (Cop1 + Co) were used as positive controls, and empty vector (pDES32) transformants were used as negative controls. (C) Representative images show the BiFC assay results using *N*. *benthamiana* epidermal cells. The split bZIP protein (bZIP-YN+bZIP-YC) was used as a positive control. YFP fluorescence generated by protein-protein interaction was detected 2 days after infiltration. Scale bars = 50 μm. (D) Co-IP between *C*. *annuum* eEF1s and PVX TGBp1 proteins. Anti-HA-agarose beads were used for immunoprecipitation (IP). Immunoblotting (IB) was performed with anti-HA for elongation factors and anti-FLAG for PVX TGBp1 proteins. GFP-FLAG was used as a negative control.

It has been reported that *eEF1A* and/or *eEF1Bs* (*β* or *γ* subunit) are involved in multiplication of several RNA viruses including TBSV, TMV, TuMV, *West Nile virus* [10, 15–17, 19]. In addition, synergistic effects of *eEF1A* and *eEF1Bγ* were found in TBSV replication in yeast [19, 36, 37]. Therefore, we tested whether pepper eEF1B subunits (α, β and γ) interact with eEF1A using yeast two-hybrid analysis. The construct for eEF1A was co-transformed into yeast (MaV203) with those for each subunit of eEF1B individually. Growth of the transformed yeast cells was assayed on synthetic complete medium (SC) lacking leucine (Leu), tryptophan (Try), histidine (His) and containing 10 mM 3-AT (SC-Leu-Try-His+3-AT). Interaction signals were detected in co-transformations including eEF1Bα or eEF1Bβ, but very weak signal was detected with eEF1Bγ for dilution factor 1.0 and 0.1 (Fig 4A). We additionally performed chlorophenol red-β-D-galactopyranoside (CPRG) assay to quantitatively measure these interactions, but no significant differences were detected (data not shown). It might be due to weak interactions between eEF1A and eEF1Bs in yeast cells. The weak signals for dilution factor 1.0 and 0.1 might be resulted from high cell density of yeast transformants. These results are consistent with previous studies showing that rice eEF1Bα and eEF1Bβ interact with eEF1A and that there is no direct interaction of eEF1Bγ with eEF1A [4]. We further tested whether eEF1A and eEF1Bβ interact with PVX RdRp, triple gene block protein1 (TGBp1), 2 (TGBp2), 3 (TGBp3), or CP using yeast two-hybrid analysis. TGBp1 of PVX strongly interacted with both eEF1A and eEF1Bβ, but the other proteins of PVX showed very weak or no interaction with eEF1A and eEF1Bβ (Fig 4B). Because very weak signals were detected in PVX TGBp2 with eEF1A or eEF1Bβ in yeast two-hybrid assay, we additionally performed bimolecular fluorescence complementation (BiFC) assays to verify these interactions. The N- or C-terminal half of yellow fluorescent protein (YN or YC) was fused with the eEF1A, eEF1Bβ or PVX TGBps (TGBp1, 2 or 3). Fluorescent signal was detected in the leaves coexpressing eEF1A-YC and PVX TGBp1-YN, or eEF1Bβ-YN and PVX TGBp1-YC as well as those with eEF1A-YC and eEF1Bβ-YN (Fig 4C). And no fluorescence was detected in the leaves coexpressing with other TGBps (TGBp2 or TGBp3) as well as in empty vector control (Fig 4C and S1 Fig). To confirm this interaction, HA-tagged eEF1A, HA-tagged eEF1Bβ, and FLAG-tagged PVX TGBp1 were transiently co-expressed in *N*. *benthamiana* leaves and co-immunoprecipitation (co-IP) was performed using anti-HA agarose beads. As shown in Fig 4D, FLAG-tagged PVX TGBp1 was co-immunoprecipitated with HA-tagged eEF1A and eEF1Bβ. Collectively, these results indicate that eEF1A and eEF1Bβ interact with PVX TGBp1.

### Identification of the Region of eEF1Bβ Important for Binding eEF1A and PVX TGBp1

**Fig 5:**
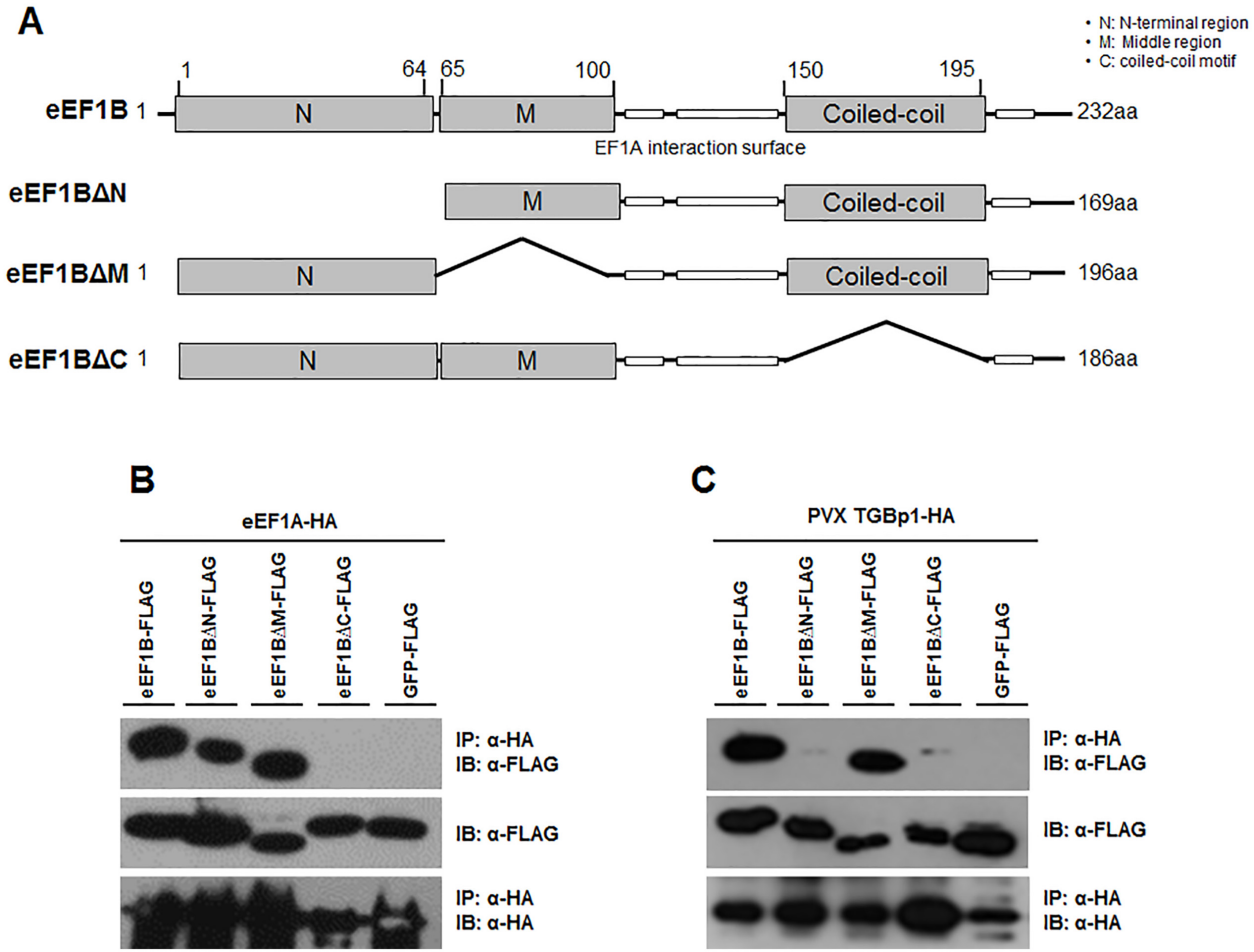
Identification of binding region of eEF1Bβ for eEF1A and PVX TGBp1. (A) Diagram showing the tested recombinant truncated variants of eEF1Bβ. eEF1BΔN, eEF1BΔM, and eEF1BΔC constructs have deletions in N-terminal, middle, and C-terminal region, respectively. White boxes indicate the well-conserved eEF1A interaction surface. ‘O’ and ‘X’ in the table indicate interaction and no interaction between eEF1B variants and eEF1A or PVX TGBp1, respectively. (B) Co-IP between deletion mutants of eEF1Bβ and eEF1A. (C) Co-IP between deletion mutants of eEF1Bβ and PVX TGBp1. For co-IP, anti HA-agarose beads were used for immunoprecipitation (IP). Immunoblotting (IB) was performed with anti-FLAG for eEF1Bs and anti-HA for eEF1A or viral proteins. GFP-FLAG was used as a negative control.

To determine which region of eEF1Bβ is important for the interaction with eEF1A and PVX TGBp1, we constructed deletion mutants of eEF1Bβ (Fig 5A), including those lacking the N-terminal region (eEF1BβΔN, deleted in amino acids 1–64), middle region (eEF1BβΔM, deleted in amino acids 65–100), or the C-terminal region (eEF1BβΔC, deleted in amino acids 150–190). The deleted C-terminal region is expected to have a coiled-coil motif (COILS program, http://embnet.vital-it.ch/software/COILS_form.html), which in EF-Ts, the bacterial counterpart of eEF1B, is important for interaction with Qβ viral RdRp [38]. For co-IP, eEF1Bβ and its deletion mutants were FLAG-tagged, and eEF1A and PVX TGBp1 were HA-tagged. The FLAG-tagged and HA-tagged constructs were co-expressed in *N*. *benthamiana*. As shown in Fig 5B, eEF1Bβ, eEF1BβΔN, and eEF1BβΔM were co-immunoprecipitated with eEF1A but eEF1BβΔC was not. In addition, eEF1Bβ and eEF1BβΔM were co-immunoprecipitated with PVX TGBp1 but eEF1BβΔN and eEF1BβΔC were not (Fig 5C). A negative control, GFP-FLAG, did not co-immunoprecipitate with eEF1A nor with PVX TGBp1 (Fig 5B and 5C). These results indicate that both the N- and C-terminal regions of eEF1Bβ are involved in the interaction with PVX whereas only the C-terminal (coiled-coil) region is important for the interaction with eEF1A.

## Discussion

### Characterization of eEF1B Complex Subunits in Plants

In this study, we classified each subunit of eEF1B in several plant species using subunit-specific motif information and phylogenetic analysis (Fig 1). One copy of *eEF1Bα* and two copies of *eEF1Bβ* and *eEF1Bγ* were found in pepper as in rice and tomato (Table 1). In pepper, *eEF1Bα* and the two copies of *eEF1Bβ* shared 57% and 59% similarity at the amino acid sequence level, respectively (Table 2). In general, eEF1Bα and eEF1Bβ from the same species show around 60% similarity at the amino acid level, with the highest similarity in the C-terminal domain (from amino acids 116 to 224 as numbered in the human eEF1Bα sequence), which corresponds to the nucleotide exchange and eEF1A interaction domain [4, 5]. The N-terminal domains of the eEF1Bα and eEF1Bβ are more divergent, although in both proteins this domain binds the structural protein eEF1Bγ [4, 7]. Plant *eEF1Bα* complements yeast *eEF1Bα* mutants whereas plant *eEF1Bβ* fails to do so [39], suggesting that plant *eEF1Bα* and *eEF1Bβ* have unique functions. In this study, we showed that PVX accumulation was reduced in the *eEF1Bβ*-silenced plants but not in the *eEF1Bα*-silenced plants (Fig 3), further supporting their unique functions. eEF1Bγ is universal in the eukaryotic kingdom and is encoded by more than one gene in most species [4]. Two eEF1Bγ homologs were found in Arabidopsis, rice, tomato, and pepper (Table 1). We found that PVX accumulation was reduced in the *eEF1Bγ*-silenced plants (Fig 3). There is no direct interaction of eEF1Bγ with eEF1A in human [4]. Similarly, pepper eEF1Bγ did not interact with pepper eEF1A in this study (Fig 4A). The N-terminal domain of eEF1Bγ was identified as interacting with eEF1Bα and eEF1B*δ* in human [4]. Therefore, eEF1Bγ appears to serve as a scaffold for the different subunits in the eEF1B complex as well as functioning in protein biosynthesis [4, 7]. In addition, eEF1Bγ plays cellular roles in RNA-binding, vacuolar protein degradation, oxidative stress responses, intermediate filament interaction and calcium-dependent membrane-binding [4, 19, 20, 40–43].

### Possible Roles of eEF1s in Virus Infection

As a multifunctional protein, the TGBp1 (TGB25K) has the activity of RNA helicase that is involved in virus movement, promotes translation of viral RNAs, suppresses RNA silencing, and increases plasmodesmal size exclusion limits for virus cell-to-cell movements [44]. Recently, it was reported that PVX TGBp1 organizes the PVX ‘X-body’, a virally induced inclusion body, by remodeling host actins and endomembranes [45]. Furthermore, TGBp1 mediates insertion of the PVX coat protein, which probably incorporates into virions, into plasmodesmata and might be related to virus movement [25]. In this study, we showed that eEF1A and eEF1Bβ interact with each other and with PVX TGBp1 (Fig 4). This suggests that eEF1A and eEF1Bβ could be involved in local and/or systemic virus movement. Since protein contents and the organized nature of the cytoplasm restrict diffusion of large molecular complexes, movement of virus-induced vesicles is likely to require cytoskeletal elements [46]. It is known that virus-induced vesicles containing virus replication complex are mobile and align with microfilaments [16, 46–49]. In eukaryotes, eEF1A has an actin-binding and -bundling function [50]. The interaction between eEF1A and actin is conserved among species from yeast to mammals. This interaction, which occurs independently of the translation elongation function of eEF1A, has been assigned to domains II and III of eEF1A [51, 52]. In addition, the usual assumption is that eEF1B*γ* serves to anchor the eEF1B complex to the membrane or the cytoskeleton [4]. Based on the overlapping function of eEF1A and eEF1B in cytoskeleton and virus protein binding, we speculate that eEF1A and eEF1B could act as an intermediator between plant virus multiplication complexes and microfilaments.

We also found that *eEF1Bγ*-silenced plants had significantly reduced accumulation of PVX (Fig 3). Previous research suggested that eEF1B*γ* has a synergistic role with eEF1A in TBSV replication in yeast, possibly via stimulation of the proper positioning of viral RdRp [19]; eEF1B*γ* binds to the TBSV replicon RNA and is a component of the virus replication complex [19]. It is possible that eEF1Bγ is involved in virus replication via directly interacting with viral RNA. Further investigation will be necessary to delineate the roles of eEF1B*γ* in RNA virus infection, for instance, by testing the interaction between eEF1B*γ* and PVX genomic RNA.

### Virus Multiplication Complex and Virus Resistance

Physical interactions between host factors and viral components are required for viral susceptibility. Naturally occurring mutations in host factors have been reported to disrupt interactions with viral proteins and confer resistance to several RNA viruses [53–55]. Similarly, cellular-level resistance may be induced by creating dominant-negative mutants or silencing targeted host genes in transgenic plants [53]. For example, overexpressing an engineered recessive resistance allele encoding eIF4E in transgenic tomato plants resulted in a highly resistant phenotype to the potyvirus *Tobacco etch virus* [56], and knock-down of both *eIF4E1* and *eIF4E2* induced broad spectrum resistance against potyviruses in tomato [57].

In the Qβ replicase complex, the Qβ virus RdRp forms a replicative complex with EF-Tu (the bacterial counterpart of eEF1A) and EF-Ts (the bacterial counterpart of eEF1B). Additionally, EF-Tu and EF-Ts tightly interact with each other [38, 58]. The EF-Tu and EF-Ts binary complex maintains the structure of the catalytic core crevasse of RdRp through hydrophobic interactions between the finger and thumb domains of the RdRp and domain-2 of EF-Tu and the coiled-coil motif of EF-Ts, respectively [38, 59]. Moreover, mutation or deletion of the amino acid residues in domain 2 of EF-Tu and deletion of the coiled-coil motif in EF-Ts reduced the complex formation and the expression of the Qβ replicase [58].

In this study, we demonstrated that *eEF1A*, *eEF1Bβ* and *eEF1Bγ* have an effect on PVX infection (Fig 3 and S1 File) and the eEF1A and eEF1Bβ proteins interact with the same viral protein of PVX (Fig 4B). We also found that eEF1A and eEF1Bβ interact with each other (Fig 4A). Therefore, we suggest that PVX resistance could be engineered by disrupting interaction between eEF1A or eEF1Bβ and PVX TGBp1. However, silencing of *eEF1A* induced severe developmental defects (Fig A in S1 File). To develop virus-resistant plants using eEF1Bβ, it will be necessary to identify amino acid residues of eEF1Bβ that are important for binding PVX TGBp1 but do not affect plant growth and development.

## Supporting Information

S1 File Effect of silencing *eEF1A* on virus infection in *N*. *benthamiana*. Phenotype of *eEF1A*-silenced *N*. *benthamiana* at 13 or 20 dpi (Fig A). RT-PCR of *eEF1A* in VIGS-treated plants. The expression levels were determined by semi-quantitative RT-PCR at 13 dpi. *Actin* was used as a standard control (Fig B). Accumulation levels of PVX in *eEF1A*-silenced plants. Accumulation of PVX in the inoculated and uninoculated leaves was tested by DAS-ELISA 7 days after PVX infection. This time point corresponds to 20 days after TRV agroinfiltration. Three independent plants were tested for treatment. The error bars indicate standard error. Asterisks denote significant differences between TRV::00 and *eEF1A*-silenced plants (unpaired *t*-test: **p <0.01(Fig C)).
(TIF)

S1 Fig Confocal microscopy images used as negative controls for BiFC assays. The co-transformed with empty vectors (YN or YC) were used as negative controls. Upper channel showed YFP images and lower channel showed light images. Scale bars = 50 μm.
(TIF)

S1 Table Primer list for this study. (DOCX)
