# Supplementary figures and images for: Plant Translation Elongation Factor 1Bβ Facilitates Potato Virus X (PVX) Infection and Interacts with PVX Triple Gene Block Protein 1
Source: PLoS One. 2015 May 28;10(5):e0128014. doi: 10.1371/journal.pone.0128014 (PMC4447259; doi:10.1371/journal.pone.0128014)

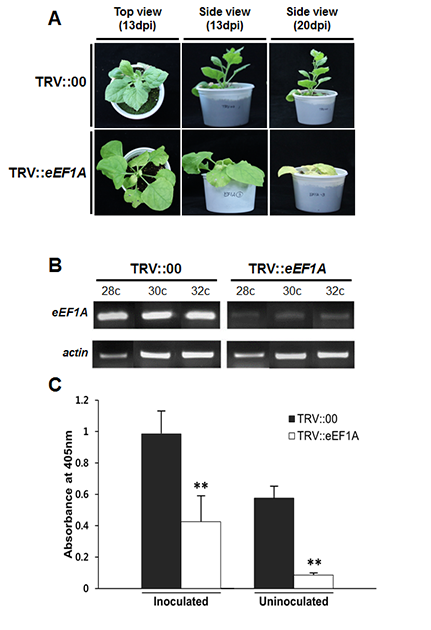

Supplement: S1 File — Phenotype of eEF1A-silenced N. benthamiana at 13 or 20 dpi (Fig A). RT-PCR of eEF1A in VIGS-treated plants. The expression levels were determined by semi-quantitative RT-PCR at 13 dpi. Actin was used as a standard control (Fig B). Accumulation levels of PVX in eEF1A-silenced plants. Accumulation of PVX in the inoculated and uninoculated leaves was tested by DAS-ELISA 7 days after PVX infection. This time point corresponds to 20 days after TRV agroinfiltration. Three independent plants were tested for treatment. The error bars indicate standard error. Asterisks denote significant differences between TRV::00 and eEF1A-silenced plants (unpaired t-test: **p <0.01(Fig C)). (TIF) [file pone.0128014.s001.tif]

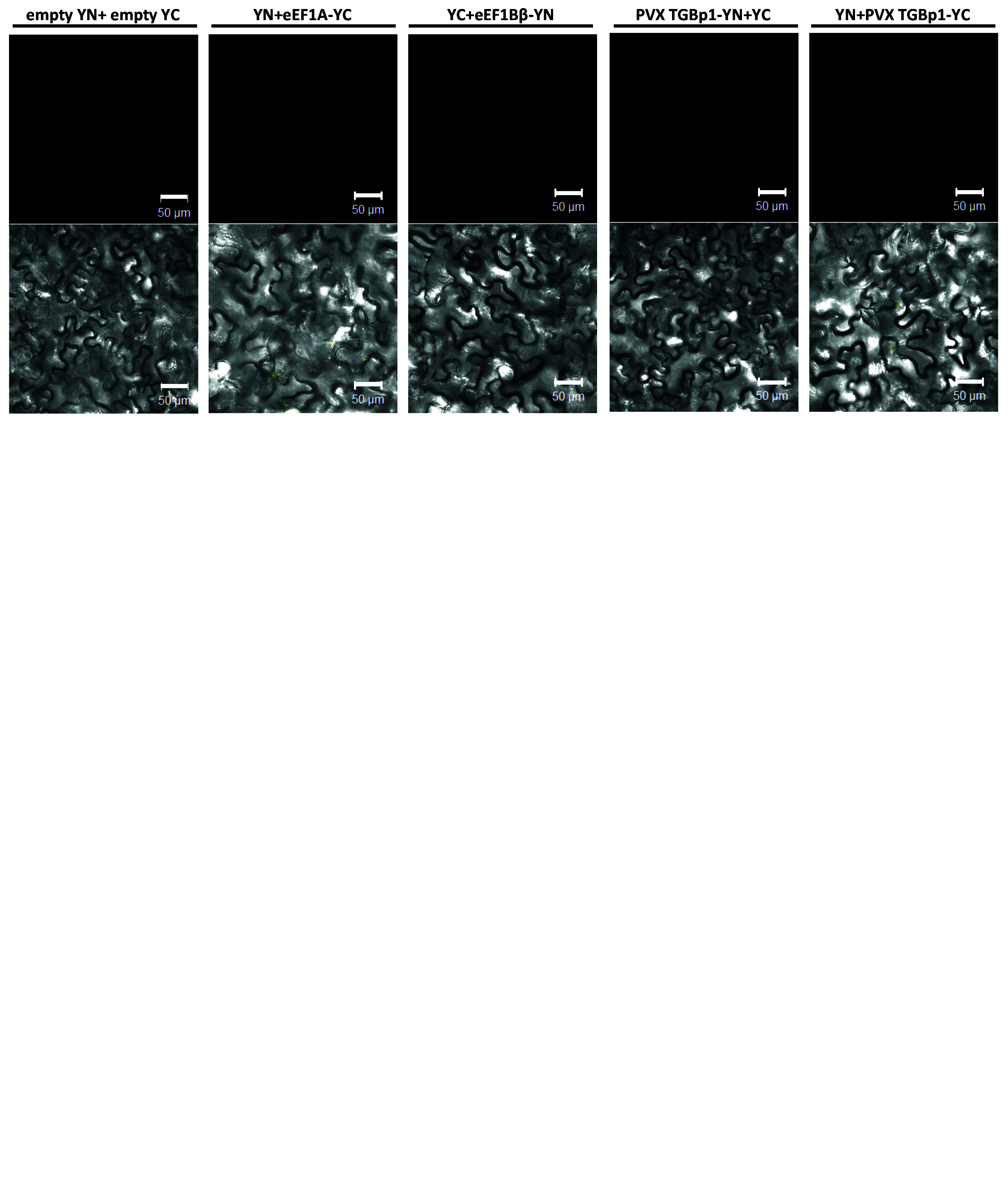

Supplement: S1 Fig — The co-transformed with empty vectors (YN or YC) were used as negative controls. Upper channel showed YFP images and lower channel showed light images. Scale bars = 50 μm. (TIF) [file pone.0128014.s002.tif]
