# Supplementary material for: Plant Translation Elongation Factor 1Bβ Facilitates Potato Virus X (PVX) Infection and Interacts with PVX Triple Gene Block Protein 1
Source: PLoS One. 2015 May 28;10(5):e0128014. doi: 10.1371/journal.pone.0128014 (PMC4447259; doi:10.1371/journal.pone.0128014)
Supplement: S1 Table — (DOCX) [file pone.0128014.s003.docx]

**SUPPORTING INFORMATION**

**S1 Table. Primer list for this study.**

| **Name** | **Sequence (5’ → 3’)** | **Application** |
| --- | --- | --- |
| eEF1A-LIC-F | CGACGACAAGACCCTTTGACAAGCGTGTTATTGAG | TRV-LIC (VIGS) |
| eEF1A-LIC-R | GAGGAGAAGAGCCCTAAAGGTCACAACCATACCAG | TRV-LIC (VIGS) |
| eEF1B alpha-LIC-F | CGACGACAAGACCCTACACTGAATCTGGTCTCA | TRV-LIC (VIGS) |
| eEF1B alpha-LIC-R | GAGGAGAAGAGCCCTTTGATGCAAGTTTAGCAG | TRV-LIC (VIGS) |
| eEF1B beta-LIC-F | CGACGACAAGACCCTTCACTGTGTATTCATCTC | TRV-LIC (VIGS) |
| eEF1B beta-LIC-R | GAGGAGAAGAGCCCTTCAGCAGCAGGAGGAGTT | TRV-LIC (VIGS) |
| eEF1B gamma-LIC-F | CGACGACAAGACCCTGGCTTTACTCCAACACC | TRV-LIC (VIGS) |
| eEF1B gamma-LIC-R | GAGGAGAAGAGCCCTGCAGAAAACCACCAAC | TRV-LIC (VIGS) |
| TRV-LIC insert-F | TGTTACTCAAGGAAGCACGATGAGCT | TRV-LIC (VIGS) |
| TRV-LIC insert-R | CAGGCACGGATCTACTTAAAGAACGTAG | TRV-LIC (VIGS) |
| 1B-α-3UTR | GCCTATCAACGAATACGTGC | eEF1Bα 3’-RACE |
| 3'-RACE (AP) | GGCCACGCGTCGACTAGTACTTTTTTTTTTTTTTT | eEF1Bα 3’-RACE |
| eEF1A-RT-F | TTTTAGTCCCTCAAGATGGGT | qRT-PCR |
| eEF1A-RT-R | AAAGGTCACAACCATACCAG | qRT-PCR |
| eEF1B alpha-RT-F | ACACCGAGTCTGGTCTCA | RT PCR, |
| eEF1B alpha-RT-R | GCAGGAGCAGCTTGGCTT | RT PCR |
| eEF1B beta-RT-F | GCATTCCAGAACCTCAACTCT | RT PCR |
| eEF1B beta-RT-R | ATATCATCCTTCGAGGCTTGG | RT PCR |
| eEF1B gamma-RT-F | GGTTTCTGGGACATGTATGA | RT PCR |
| eEF1B gamma-RT-R | ATTCATAGAGCTCCATGTC | RT PCR |
| Nb-actin-F | CCAGGTATTGCTGATAGAATGAG | RT PCR, qRT-PCR |
| Nb-actin-R | CTGAGGGAAGCCAAGATAGAG | RT PCR, qRT-PCR |
| eEF1B-del_N_F | **TCTAGA**TGTTGAGAATCTCTGGTGTA | Y2H, BiFC |
| eEF1B-del_N_R | CTCGAGTATCTTGTTGAAAGCAAC | Y2H, BiFC |
| eEF1B-del_M-F | GCACTTGCTGTTGAAGATGATGAC | Y2H, BiFC |
| eEF1B-del_M-R | AACAGCAAGTGCATCAATGTGCTT | Y2H, BiFC |
| eEF1B-del_C-F | GATGTCATTGTCGATGACTTGGTC | Y2H, BiFC |
| eEF1B-del_C-R | GACAATGACATCCAGGAGAACTGA | Y2H, BiFC |
| eEF1A-XbaI-F | **TCTAGA**ATGGGTAAGGAAAAGATTCA | Y2H, BiFC |
| eEF1A-XhoI-R | **CTCGAG**CTTTCCCTTCTTCTGGGC | Y2H, BiFC |
| eEF1Bβ-XbaI-F | **TCTAGA**ATGGCTGTTGCATTCAAC | Y2H, BiFC |
| eEF1Bβ-XhoI-R | **CTCGAG**TATCTTGTTGAAAGCAAC | Y2H, BiFC |
| PVX TGBp1-SpeI-F | **ACTAGT**ATGGATATTCTCATCAGT | Y2H, BiFC |
| PVX TGBp1-XhoI-R | **CTCGAG**TGGCCCTGCGCGGACATA | Y2H, BiFC |
| eEF1B alpha-F | ATGGCTGTAACTTTCTCAAATC | phylogenetic analysis |
| eEF1B alpha-R | TTAGTTCAGCACGTATTCGTTG | phylogenetic analysis |
| eEF1B beta-F | ATGGCTGTTGCATTCAGCAACC | phylogenetic analysis |
| eEF1B beta-R | CTAACAAAGAACTACTGATCGC | phylogenetic analysis |
| eEF1B gamma-F | ATGGCTTTGATTTTACATTC | phylogenetic analysis |
| eEF1B gamma-R | TTACTTGAAGCACTTTGCATCC | phylogenetic analysis |

Adaptor sequences for LIC (Ligation Independent Cloning) are underlined.

The restriction enzyme sites used for cloning are shown boldface.
